# The experience of mothers of autistic children with a pathological demand avoidance profile: an interpretative phenomenological analysis

**DOI:** 10.1007/s44192-025-00127-3

**Published:** 2025-01-20

**Authors:** Sam Curtis, Elizabeth Izett

**Affiliations:** School of Arts and Humanities, Edith Cowan University, Perth, WA Australia

**Keywords:** Pathological Demand Avoidance, Autism, Parent Experience, Qualitative

## Abstract

**Purpose:**

Emergent research literature has identified emotional and behavioural challenges for autistic children with a pathological demand avoidance profile. However, understanding of their parents’ experience is limited. This study aimed to explore the experience of parents of autistic children with a pathological demand avoidance profile.

**Methods:**

Semi-structured interviews were completed with ten parents of autistic children with a pathological demand avoidance profile, aged between 5 and 11 years (M = 8.5, SD = 1.90). All participants were mothers, aged 33–50 years (M = 42, SD = 5.35). To explore what meaning participants gave to their lived experience, an interpretative phenomenological analysis was conducted on interview data.

**Results:**

Four main themes were developed from the interpretative phenomenological analysis; the benefit of a shared understanding about pathological demand avoidance to the parents and their children, the power of pathological demand avoidance and the impact on families, the emotional experience of mothers, and the various ways in which they coped.

**Conclusion:**

The need for further recognition and understanding about pathological demand avoidance is emphasised through recommendations for future research. As is the need for flexible, informed, and appropriate support for demand avoidant children and their families.

**Supplementary Information:**

The online version contains supplementary material available at 10.1007/s44192-025-00127-3.

## Introduction

Pathological demand avoidance (PDA) is a term that was developed by Newson and colleagues to describe a cohort of ‘atypically autistic’ children, who came to clinical attention due to their extreme, obsessive avoidance of everyday demands [1]. Autistic people experiencing demand avoidance, and their families, identify with the term “PDA”, despite it yet being a formally diagnosable condition [2]. This demand avoidance phenomenon is understood to be driven by an anxious need to be in control and a strong intolerance of uncertainty [3], which can lead to behaviour challenges [4]. What is perceived as a demand is unique to each individual, and can range from direct demands, such as requests made by other people, to indirect demands, such as transitions, expectations, and receiving praise [5]. There is often a degree of sociability observed in PDA that is unusual for autistic children, as PDA children may engage in socially strategic behaviours to avoid demands [5]. Demand avoidance behaviours may range from more subtle forms of social control, such as excuses or engaging in fantasy and role play, to more extreme behaviours, such as drastic changes in mood, aggression, violence, and threats [1, 4–7].

PDA is not currently recognised within diagnostic classification manuals such as the Diagnostic and Statistical Manual of Mental Disorders (5th ed., text rev., DSM-5-TR) [8] or the International Statistical Classification of Diseases and Related Health Problems (11th ed.; ICD-11) [9]. However, recognition of PDA as a distinct entity is increasing within autism research and advocacy groups, including acknowledgement in practice guidelines [10–12]. Requests for PDA diagnosis by parents are also increasing [6]. PDA is mostly considered as a profile that exists within the autism spectrum [3, 13, 14], and professionals may assign a “PDA profile” to an autistic child experiencing demand avoidance [15]. Researchers have debated whether PDA should be considered as an external co-occurring condition related to heightened anxiety [16] or as a set of outcome ‘symptoms’ of other recognised conditions co-occurring with autism, such as anxiety, oppositional defiance, and conduct disorders [6]. Only one study has investigated the prevalence of PDA in autistic children using population cohort data from the Faroe Islands and suggested that one in five children with autism showed characteristics of PDA [13].

When PDA children are pushed to comply with demands, their behaviour tends to become more oppositional and extreme [7]. This is likely to create significant difficulties for their parents, who have described the pervasiveness of their child’s demand avoidance in every aspect of daily life [17, 18]. A recent study highlighted the impact of PDA children’s extreme anxiety on parental wellbeing, impacting emotional and physical health, sleep, relationships, and family dynamics [18]. Additionally, research has indicated that parents of PDA children are often blamed by professionals for their child’s problem behaviours and lack of progress [19, 20]. In one report summarising responses to an online survey, some parents of PDA children perceived that parental blame had contributed towards a deterioration of their mental health and a distrust of services [19]. Although there is a scarcity of literature on how parents can help their PDA children [21], a low arousal, low demand parenting approach is at least anecdotally effective [7].

Whilst research on the experience of parents of autistic children with PDA is limited, the extant autism literature provides some insight into their possible experience. Qualitative research has revealed that parents of autistic children face a range of difficult emotions around their child’s autism diagnosis. This includes feelings of shock, sadness, stress, grief, denial, guilt, anger, and worry [22]. Parents described coping with these emotions by accepting their child and focusing on what could be done to help them [23]. The demand of raising an autistic child means that many parents report experiencing social isolation [22, 23]. Stigma is also a common experience [24], felt more acutely in parents of autistic children with aggressive behaviour [25, 26], which may have implications for the parents of PDA children who experience parent-blaming [19]. Gore Langton and Frederickson [27] argue that negative effects may be heightened in parents of PDA children who face similar challenges to those faced by parents of autistic children, before autism became more widely understood.

Some of the challenges that PDA individuals and their families face appear to include a lack of awareness about PDA, barriers to accessing support, and parental stress which negatively impacts their well-being [18]. However, the research literature on PDA is still in its infancy, and there is only a limited understanding of the experiences of parents of PDA children. To further understand these experiences, this phenomenon must be explicitly explored. This study aimed to explore the lived experience of parents of autistic children with a PDA profile. By exploring what meaning parents make of their experiences it is hoped that service providers and professionals can be better equipped to support both parents and their children.

## Method

### Design

A qualitative research method was used for this study, with an interpretative phenomenological analysis (IPA) adopted to analyse the data and explore the meaning that participants gave to their lived experience [28]. This study was conducted from an interpretivist epistemological position, which holds that reality is subjective, socially constructed and a composite of multiple perspectives. In this approach, the researcher is acknowledged as having an active meaning making role. Smith and Osborn [29] describe this as a two-stage interpretation process. The participants are trying to make sense of their world; and the researcher is trying to make sense of the participants trying to make sense of their world. Acknowledging the active role of the researcher, existent PDA literature was reviewed, and the researcher participated in PDA training provided by a clinical psychologist with expertise in PDA. The training focused on working therapeutically with PDA clients, including education on PDA traits and therapeutic approaches.

### Procedure

Once ethics approval was obtained from the Edith Cowan University Human Research Ethics Committee (REMS NO: 2022-04029-CURTIS), recruitment material was shared in the ‘PDA Perth WA Parents Community and Support Page’ Facebook group. The recruitment material provided the researchers’ contact information and urged interested parents to contact the researchers. Participants were given the opportunity to ask questions about the study and emailed a link to a Qualtrics questionnaire, which included the participant information letter and consent form, a demographic questionnaire, and an online version of the Extreme Demand Avoidance Questionnaire (EDA-Q) [30].

With informed consent obtained, participants who provided scores equal to or above the recommended cut-off for PDA likelihood were invited for an interview. Audio was recorded using a personal audio-recording device, and transcribed verbatim. Participants were informed of their ability to stop or withdraw at any moment and invited to provide feedback immediately after the interview. Participants were then sent an email debrief sheet which included information about how to withdraw their data and access relevant support services if required. The debrief sheet also provided helpful information from key informants such as PDA resources, parent support groups, and suggestions for further research participation.

### Data analysis

Participants’ identifiable information from the demographic questionnaire and EDA-Q was linked to an identification number and stored in a password protected folder on the researcher’s computer. Audio-recordings and transcriptions needed to be linked to participants’ demographic data to allow participants time to review and withdraw their data if desired. An interpretative phenomenological analysis of the data was used following the process outlined by Smith et al. [28]. Given the idiographic nature of IPA, each transcript was analysed, in detail, before analysis of the next case. The initial transcript annotations were consolidated into experiential statements which reflected both a summary of the important words and thoughts of the participant, and the researcher’s interpretation. Connected experiential statements were then clustered into personal experiential themes. A table was formed for each individual case outlining identified personal experiential themes and experiential statements. The individual analysis of the next case only began at the conclusion of the in-depth analysis of the case prior. Once all cases were analysed, a table of group experiential themes was developed, with analysis of similarities and differences across cases.

Strategies were also implemented to ensure research quality. A bracketing process was completed prior to data collection, recording the researchers’ biases, assumptions, and preconceptions about the phenomenon in question, to increase awareness and reduce their influence on the research [31]. The researchers also acknowledged their own identities and their potential influence on data collection and analysis. The researcher SC was a male clinical psychology student in their twenties, who had professional experience with autistic children, and no personal relationships with participants, parents or autistic children with a PDA profile. The researcher EI was a female clinical psychologist academic who also works in private practice with autistic children, adolescents and their parents. She had no personal relationships with the parents or autistic children with a PDA profile included in this study. Reflexive supervision between SC and EI was used throughout data collection, to enhance researcher awareness and refine research skills [32]. Each research stage was recorded in an audit trail and reviewed periodically to ensure that a coherent chain of arguments existed from the transcript annotations to the final report [28]. A parent of a PDA child, who is a local autism community advocate and organiser of parent support groups for parents of PDA children, was involved in conceptualising the research, for community engagement in research design [33, 34].

### Measures

#### Semi-structured interviews

Data was collected using semi-structured interviews with participants, in their homes, online using Microsoft Teams, or in a centrally located clinical office in Perth, Western Australia, according to the parents’ preference. Open-ended questions were asked from the interview guide such as “how has PDA affected your relationship with your child?” and “what effect has PDA had on you?” (Supplementary file 1). The interview guide was informed by consultation with a community advocate and parent of a PDA child, and a clinical psychologist with expertise in PDA, to ensure that it was an appropriate tool for exploring the parents’ experience. The interviews were relatively unstructured, allowing conversation to flow from topics raised, with minimal prompts such as “can you tell me more about that?” used when required. Care was taken not to lead participants to answers, letting them tell their story in their own words.

#### Extreme demand avoidance questionnaire (EDA-Q) [30]

The EDA-Q is a freely available 26-item questionnaire developed for research, to measure PDA behaviours in children. The EDA-Q was administered to parents prior to interview to ensure participants were experiencing the phenomenon in question. Parents were asked to rate the extent to which features were consistent with their child’s behaviour over the last 6 months. Each item was rated on a 4-point Likert scale ranging from “not true” to “very true”. EDA-Q items were developed based on criteria described by Newson et al. [1], and PDA items from the Diagnostic Interview for Social and Communication Disorders [35]. Item examples include “obsessively resists and avoids ordinary demands and requests” and “has difficulty complying with demands unless they are carefully presented”. The recommended composite cut-off score for elevated likelihood of PDA is 50 for children aged 5 to 11 years, as this score maximises sensitivity and specificity for distinguishing individuals at risk of exhibiting PDA characteristics from case comparison groups [30]. Participants that returned EDA-Q scores below the recommended cut-off were excluded from interview, unless they were able to provide documented evidence of a clinically recognised PDA profile. Currently, there is no official diagnostic process for PDA in Australia. However, clinicians may use a combination of developmental history interviews, observations, and psychometric tools such as the EDA-Q, to assign a “PDA profile” whilst diagnosing autism [15]. The EDA-Q is suggested to demonstrate good content validity and internal consistency (α = 0.87) [30].

### Participants

Purposive sampling was used to recruit English speaking parents of autistic children with PDA consistent behaviours, aged 5 to 11 years. Ten participants from Perth, Western Australia aged between 33 and 50 years (M = 42, SD = 5.35) were included in this study. All participants were female and biological mothers to their PDA child. The age of participants’ PDA children ranged from 5 to 11 years (M = 8.5, SD = 1.90), with four children being female and six being male. The researchers selected this required age range to narrow the focus to the experience of parents of primary school aged children. The plausible differences in experience between parents of PDA children and PDA adolescents may have complicated data analysis and interpretation. Additionally, a longitudinal population study of PDA in the Faroe Islands suggested that some PDA signs may be specific to earlier developmental phases, as only one out of nine participants with indications of a PDA diagnosis reported having sufficient remaining symptoms to “qualify” for a diagnosis at age 15–24 years [13].

Participants were recruited from the ‘PDA Perth WA Parents Community and Support Page’ Facebook group. Thirty-seven parents registered their interest in participating in the study. Twenty-five participants completed the demographics questionnaire and EDA-Q. Three participants returned EDA-Q scores below the recommended cut-off score of 50 and were subsequently excluded from interview, as they did not alternatively provide documented evidence of a clinically recognised PDA profile. One participant was also excluded as their child had not been diagnosed with autism. Twenty-one participants were offered an interview. However, 11 participants either stopped contacting the researcher or did not attend their arranged interview. The final sample of 10 participants was deemed sufficiently representative of the target population in their specific context, whilst also allowing for a detailed account of individual experiences [28]. Table 1 presents a summary of participant demographic data, EDA-Q scores, and diagnostic information. Participants were provided with pseudonyms to protect their anonymity.

**Table 1:** Participant Demographic Data

Pseudonym	Age	Child’s age	Child’s identified gender	Other family members and age	Autism diagnosis	PDA profile	EDA-Q score
Justine	36	7	Female	Sister (10), sister (9)	Yes	Yes	73
Sharon	41	8	Male	Father (40), sister (11)	Yes	Yes	50
Jessica	38	8	Male	Father (40), sister (12)	Yes	Yes	63
Debra	43	7	Male	None	Yes	Yes	55
Leslie	49	9	Female	Mother (51), sister (11)	Yes	Yes	58
Janet	42	9	Female	Father (43), brother (7)	Yes	Yes	50
Alison	33	5	Male	Father (33), sister (8), brother (7), sister (4)	Yes	Yes	53
Cheryl	43	11	Female	Father (43), sister (10), half-sister (17)	Yes	Yes	59
Lisa	45	11	Male	Father (52), brother (8)	Yes	Yes	67
Simone	50	10	Male	Father (44)	Yes	No	56

## Results

Four main overarching themes were developed from the IPA data analysis, which are summarised in Table 2. The first theme described the benefit of shared understanding about PDA to the parents and their PDA children. The second theme explored the power of PDA and the impact that power had on families. The third theme described the emotional experience of parents, and the effect that parenting challenges had on them. Finally, the fourth theme highlighted the strength and resilience of the parents through the various ways in which they coped.

**Table 2:** Superordinate and Subordinate Themes

Superordinate themes	Subordinate themes
The Benefit of Shared Understanding About PDA	Limited Recognition, Understanding, and Support
	Receiving Judgement and Blame
	Understanding the Child to Support Them
	Forced Advocacy Role
The Power of PDA	Demanding Relationship
	Isolation
	Difficulty Attending to Competing Family Needs
	Grieving Expectations of Normality
Emotional Toll	“Capacity is Always at Zero” (Justine)
	Empathising and Mentalising PDA Child
	Parental Doubt
	Resenting PDA
Coping	

### Theme 1: the benefit of shared understanding about PDA

#### Limited recognition, understanding, and support

Parents described their experiences of the limited awareness, recognition and understanding of PDA amongst others. This was experienced in a variety of ways, including encounters with health professionals.

“Nobody knew anything about it, even talking to GP’s or paediatricians, and the psychologists who had been in the first school.” (Lisa).

Parents reported that their challenges parenting their PDA child were misunderstood.

“I think misunderstood is probably you know, important because, again, I think if I had a child with cerebral palsy, everyone would know what that was. But it's really difficult because I don't think people expect us to be under as much pressure as we are.” (Cheryl).

This comparison of PDA to cerebral palsy refers to the implicit understanding that others have of the challenges one might face in parenting a child with a recognisable, visible disability, which is missing for parents of PDA children. This limited understanding meant that it was difficult to get support.

“We don't have other support, … we're the only ones, and you can't reach out more cos other people don't know how to manage them.” (Cheryl).

A lack of practical and PDA-informed support made some parents angry.

“I think I was searching and hopeful that there was a solution and that there was someone to help. And then I got pissed off and angry that there wasn't anyone to help.” (Leslie).

The detrimental impacts of limited understanding about PDA were not restricted to a lack of support. Justine and Lisa reported that their PDA children experienced harmful practices from professionals that did not understand how to support their child and responded with either aggression or by forcing compliance.

“Some of these behaviours that (PDA child) had, you know, when he's distressed and… spitting and throwing… he was just a really distressed little human. And so at school, unfortunately, this became met with aggression. And by a staff member, who assaulted (PDA child) on three separate occasions.” (Lisa).

Interacting with professionals who do not understand PDA can be counterproductive and demoralising. However, when dysregulated behaviours are responded to with control or aggression, there can be severe consequences for the PDA child and their parent.

“They would take the mentality of oh you just need to force them… the kid would walk away from their sessions and be traumatised.” (Justine).

#### Receiving judgement and blame

Parents reported being blamed for their child’s challenges, including being judged for their ‘lenient’ parenting.

“But when you're dealing with things like the NDIS, and other health professionals, like paediatricians, and things like that, that are old school, they tend to put the blame on you, like as me as a parent. I'm too lenient. I don't put in the work. It's because I'm too soft. And we're not following through with things and strategies, which is a load of crap. Because it's all we're doing, is that, yeah, so it was kind of insulting to me and demeaning of my character and like, my position as being his mum.” (Jessica).

In this example, Jessica refers to the National Disability Insurance Scheme (NDIS) which is a program in Australia that provides funding and support to people with disabilities and their families. Jessica spoke about her interactions with “old school” health professionals that do not understand PDA. As a result, Jessica felt that her parenting was blamed for their anxious child’s dysregulated behaviour. The insult to Jessica’s identity as a mother made her angry.

Sharon simply encapsulated the misunderstanding and judgement that parents experienced in the following example, seemingly feeling invalidated.

“PDA just seems to be this weird, fake thing that bad people have for people who can't parent well.” (Sharon).

#### Understanding the child to support them

Many parents reported embarking on a self-education journey. Due to difficulty understanding their child, parents would search for answers and eventually discover PDA.

“The more I learn about PDA, the more it helps my parenting journey. And it helps me understand my PDA child as well, because I felt I just didn't really understand her.” (Janet).

Simone described how she discovered PDA and the helpfulness of that discovery in this example.

“I fell in a bit of a heap. So, then I finally did start joining two little Facebook groups on autism and started listening in on things. And then one day, I read something about PDA. And, I think most PDA parents will probably say this, they, they hear about PDA, and all of a sudden, everything makes sense. And, you know, I could have written what this parent had written. So then I, someone mentioned something about a PDA group. So then I joined that. And from that moment, it's like, okay, this is what it is.” (Simone).

Parents described a “light-bulb moment” (Janet) when discovering PDA, which helped them to understand their child and how to support them. The similarity of experience between parents of other PDA children also helped parents to feel validated and connected through shared understanding about PDA.

“A really good, magic inbuilt support, because they just sort of get it.” (Sharon).

#### Forced advocacy role

Not only was it imperative for parents to understand their PDA child, but they also described a burden to educate others about PDA and to advocate for their children too.

“She's obsessed with me because I give her what she needs. And until other people give her what she needs, she won't be un-obsessed with me and rely on them. And so my job is to make other people like that, which is ridiculous, but then who else is gonna do it, the paediatrician’s not gonna come out and teach (her partner) how to do it. They're not going to go and educate my parents. So I have to do all that.” (Cheryl).

Here, Cheryl described the closeness of her relationship with her child as she can understand her, and accommodate her need for autonomy and control. To alleviate some of that reliance on her and increase the child’s support, the parent is then required to educate other people on how to accommodate their PDA traits too. The responsibility to advocate for their child was difficult for some parents.

“The school and stuff like that used to always be like, I'm gonna burst into tears if I go in there and try and fight for my kid.” (Debra).

### Theme 2: the power of PDA

#### Demanding relationship

Parents portrayed a parent–child relationship that demanded a lot from them to accommodate their child to help them function in their everyday life. They described adapting their parenting approach to afford their child as much autonomy and control as possible, as this helped their child to stay emotionally regulated.

“So, (PDA child) takes the lead of our family. And that works because it keeps things neutral, and calm.” (Jessica).

Parents described the difficulty of that dynamic in their relationship with their child, as the traditional perspective of parent–child relationships places power with the parents.

“It's hard to let go of the control to your child, you know, when you so badly want to be the parent and to have this ideal way of kind of, you know, parenting and doing things and hopefully guiding them and teaching them in life. But the more that you try and guide and teach the more it sets them off.” (Janet).

As parents were able to understand their child and meet their emotional needs through affording them autonomy and control, they described an intensely close parent–child relationship. Parents noted a greater than expected need in their PDA children for coregulation from their parents.

“She's unable to independently self-regulate, like she solely relies on me. So, because everything in her world is so intense, she needs to be by my side 24/7”. (Janet).

“I'm like his wheelchair, you know, for a person who's got a physical handicap. I'm like, yes, I'm that for him. So, I know how much he needs me.” (Lisa).

Parents described the responsibility to constantly attune to and accommodate their child’s emotional needs as demanding. The term “safe place” was often used to describe the parent–child relationship, as a place for their child’s overwhelming emotions to be expressed safely.

“I'm his safe place. So I see the best and I see the worst…I'm the one he wants to cuddle when he's upset. But then I'm also the one that he'll shout at and say he hates when he's feeling really dysregulated.” (Simone).

This safe place for the child could, at times, be unsafe for the parents, who described experiencing physical assaults from their dysregulated PDA child.

“We were getting to the point where we left our motorbike jackets on and our helmets on (to) stop from being hurt so much.” (Leslie).

Parents’ descriptions of the demanding relationship with their child ranged from feeling like “prison” (Jessica), a “slave” (Debra), or a “domestic violence relationship” (Lisa). These descriptions depicted a sense of entrapment and powerlessness that parents experienced in their parent–child relationship. However, whilst this demanding relationship often required parents to prioritise their child’s needs over their own, parents were accepting of making that sacrifice.

“Having to find that balance between doing what's right for them, what's right for me, it can be tricky. And more often than not, it would be me, my needs and wants, that I will more than happily just go, doesn't matter, and get into burning out.” (Justine).

#### Isolation

The demanding parent–child relationship meant that maintaining or forming relationships was difficult for many parents. This is captured in this example of Janet talking about her hope for re-partnering.

“You know, I would maybe eventually hopefully think I could have another partner one day, but I don't feel confident yet in being able to manage that because she needs me full time.” (Janet).

This also applied to other relationships, as parents described losing friendships too.

“Friends have kind of dropped off by the wayside because you have to cancel at the last minute.” (Leslie).

Families with PDA children often spent a lot of time at home, as this helped the PDA child to stay regulated.

“Sometimes you'll plan to do something, but it's just not going to happen. So, we'll just stay home in the home bubble.” (Sharon).

The unpredictability of their child’s dysregulation and the reliance on the parents for support was described as isolating.

“Definitely struggled in terms of feeling alone in the battle.” (Sharon).

#### Difficulty attending to competing family needs

The demands of raising a PDA child meant that there was limited time for parents to attend to their relationships with other family members. It was difficult for parents to manage attending to competing needs.

“How can I make this situation better for him (PDA child). But it's also hard when it's affecting other children as well…So I just, I kind of have to find that balance between all the kids to try and make it work. And sometimes, sometimes it works and sometimes it doesn't.” (Alison).

Prioritising their PDA child was a difficult dilemma for some parents, who felt guilty and ashamed of their limited capacity to be there for their other children.

“What a crappy mum I’ve been to my other child having to choose. That is a phenomenal problem I have daily.” (Cheryl).

Parents also felt that their relationship with their partner could be neglected as they needed to prioritise caring for their PDA child.

“We've had to put our relationship on the shelf to sort of keep our child going.” (Sharon).

#### Grieving expectations of normality

The demands of parenting a PDA child had meant that being a parent looked quite different to how they had imagined. Parents spoke about grieving their “hopes and dreams” (Jessica) for their family. Parents often compared themselves to other families, and what people might take for granted in ‘normal’ life.

“Most parents, at the end of the day… have an hour to watch crap on TV, do some jobs or read a book or whatever, like. I don't get that window.” (Sharon).

There were also descriptions of typical experiences that parents had sacrificed.

“So that's limited me working. Yeah, doing anything, my friends, family trips and things like that. And because it's all child led.” (Jessica).

Loss was often attributed to giving their child autonomy and control, and prioritising their child over other aspects of life. Additionally, parents described grieving their expectations of what their relationship with their child would be like.

“That’s another thing, you don't know that you're gonna get hit by your kid and spoken to the way you are spoken to by your kid.” (Debra).

Parents wanted to be experienced by their children as “a parent, not as someone to scream at.” (Jessica).

### Theme 3: emotional toll

#### “Capacity is always at zero” (Justine)

Parents described the significant emotional toll that raising a PDA child had on them, often feeling exhausted.

“Yeah, a lot of the time drop off at school is like, I've run a marathon by nine o'clock in the morning.” (Debra).

Parents’ reported exhaustion seemed to be due to their difficulty supporting their PDA child. This manifested in stress and anxiety for parents who described constantly considering how to best manage their child’s needs.

“I have anxiety, constant anxiety, like this anticipatory anxiety. Like, I wake in the morning ready for, you know, not knowing what's going to happen and what direction the day's gonna take with her and what she needs of me.” (Janet).

Janet’s experience of stress and anxiety appeared to result from the unpredictability of her child’s dysregulation combined with the intense demands of the parent–child relationship. 

Parents’ emotional distress was often mentioned in the context of the burden of their caregiving responsibility, and their limited capacity to support their child and to implement professional advice or parenting interventions.

“So, every time consults with psychs or with the behaviour support, whatever, it's all information for me to download and take on and to implement and then redistribute … I find that exhausting and I don't actually have capacity to do that myself and half the time … It's kind of in one ear and out the other because I actually, I don't have the mental capacity.” (Lisa).

Both Debra and Cheryl felt as though the stress of parenting their PDA child had contributed to the development of physical illness.

“My body's attacking itself, because I'm not getting enough rest.” (Cheryl).

#### Empathising and mentalising PDA Child

Parents expressed their curiosity about their child’s experiences and empathy for their difficulties. There were clear attempts to mentalise their PDA child.

“Yeah, it's sad to think that he might, you know, miss out on just bits and pieces that you know, we do because of this (PDA)… you could see that conflict, I want to do it, but no, I'm not going to do it, because everyone thinks I should be doing it.” (Alison).

Parents’ knowledge of PDA could be applied to understanding their child’s internal mental states when experiencing demands. In the following excerpt, Sharon explained her perception of the impact of demand avoidance on her child’s emotions, hindering their capacity to be themselves.

“Over the summer holidays when he hasn't been in school, and we've been able to sort of be very low demand … you could see him again, you know, he's in there still. He's not just this ball of angry anxiety all the time”. (Sharon).

Sharon then described what that was like for her to think about demand avoidant anxiety hindering her child’s capacity to be themselves.

“Devastating. Because, you know, there's that little person in there. And you know he's there, somewhere. And he should be able to be himself. And, you know, the world around doesn't quite get it and let him be that”. (Sharon).

This excerpt captures Sharon’s understanding of her child’s experience in a world that fails to understand or accommodate his PDA traits. It also demonstrates the positive impact that an accommodating environment can have on a PDA child’s emotional state and functioning.

Parents described their understanding of their child’s struggles as difficult for them as well. Having awareness of their child’s suffering from their child’s perspective, took an emotional toll. Parents were also worried about the cumulative impact of being constantly dysregulated, and failing to meet others’ expectations, on their child’s developing sense of self.

“(I’m worried) that they're going to stop loving themselves and seeing themselves as valuable. And view themselves the way that others tend to… difficult, broken, wrong, a burden.” (Cheryl).

#### Parental doubt

Parents reported doubt about their parenting. Often, suggestions that their parenting had contributed to their child’s difficulties created doubt.

“I haven't tried hard enough. I'm not firm enough with him. Which led me to second guess everything I've done”. (Sharon).

Simone was angry at suggestions that caused her to doubt her parenting and impacted her relationship with her child.

“My natural instincts or my, my intuition, how to parent him. People were constantly telling me not to do that… And so I didn't, which damaged my relationship with my child. But also, actually turns out, I'm gonna swear here, I was fucking right. That, you know, like, and they made me question myself. So that makes me angry. Very angry, actually. Because it's, you know, they're not actually listening”. (Simone).

Here, Simone described not following her ‘intuition’ in parenting her child, as uninformed suggestions created doubt about her parenting approach.

Some parents experienced guilt or self-criticism about how they parent their PDA child. This was apparent when Cheryl discussed becoming angry at times when her child was dysregulated.

“I mean, I feel like I'm the worst mom.” (Cheryl).

#### Resenting PDA

The challenges of parenting a PDA child meant that sometimes parents felt resentment.

“So, you just get the feeling sometimes, like "fuck you", you know. “I do everything for you, and there's not even an acknowledgement that I'm your mum, let alone anything else”. So, you do get that. Yeah, this, you do resent it (PDA) a bit. It's hard. Because you do a lot.” (Jessica).

When Jessica described the demands of parenting her PDA child, the thanklessness of her sacrifice appeared to contribute to her resentment. Parents that shared their resentment towards their PDA child expressed guilt and shame along with those feelings.

“I didn't want to admit that (resentment), but I knew inside, and I still feel that from time to time, and that's a horrible, horrible, horrible thing to feel about your child.” (Janet).

### Theme 4: coping

Parents’ resilience and strength was apparent in their various methods of coping with the challenges of parenting a PDA child. Often, parents spoke about cherishing rare moments of independence from their child. Attending work provided welcome independence for the few parents that were able to maintain limited hours of employment.

“I go to work for a break. Yeah, most people come home for a break from work, but yep.” (Sharon).

The following passage captured the constant mental toll of parenting a PDA child and the value of taking mental breaks.

“In my head is always constant worry. Yeah, it's research, worry, therapy. It's because he's so loud as well. So he's constant noise all the time and he's constant demands. So yes, it's never quiet in there. So when I do get those times now I've gone from socialising and having a drink or, to just my own space.” (Jessica).

Some parents coped by focusing on how they could help their child, which could be motivated by anger about their challenging parenting experience.

“I don't sleep. I throw myself into reading more. I get angry.” (Cheryl).

Often, parents spoke of avoiding potentially demanding social situations to cope with the challenges of managing their child’s dysregulated response to demands.

“We don't take him for catch ups with old friends and their kids, because we just can't trust that he's not going to get dysregulated when he doesn't feel comfortable.” (Simone).

Parents spoke of being particularly grateful for the good moments and often reminded themselves of the positives of their relationship with their child.

“Celebrating the wins is so much bigger than the next kid.” (Debra).

Parents also described holding hope that life would be easier for both them and their child in the future.

“Just become a little bit more hopeful recently…. It's better to live in that place.” (Debra).

## Discussion

This study aimed to explore the lived experience of parents of autistic children with a PDA profile. This phenomenon was explicitly explored as an understanding of their parenting experience is only beginning to develop. This study contributes to the emerging PDA literature by illuminating the challenging experiences of mothers of PDA children. It is hoped that with more understanding about their experience, service providers and professionals can be better equipped to support PDA children and their families. The key findings of this study were; the benefit of a shared understanding about PDA for the parents and their PDA children; the power of PDA and the demand that places on parents and the broader impact on their families; the emotional toll of parenting a PDA child; and the various methods parents used to cope.

Consistent with previous research, one of the key findings of this study was the need for more understanding about PDA [18]. Parents spoke about their experience of interacting with professionals in education and health-care settings who had limited understanding about PDA, invariably leading to misguided advice, poor support, or harmful practices. They also reported experiencing stigma. Consistent with previous PDA literature, parents reported receiving judgement for their child’s behaviour and being blamed for their perceived leniency or poor parenting [19, 20]. Parents’ reported experience of stigma in this study has been conceptualised in research literature as affiliate stigma [36] or family stigma [37]. This stigma-by-association refers to the negative attitudes directed towards individuals due to their relationships with a stigmatised person.

Parents attributed their experience of affiliate stigma to the limited understanding about PDA amongst others. Parents felt that the ‘invisibility’ of their child’s disability made them more susceptible to affiliate stigma, as there was an absence of implicit understanding about the inherent challenges of parenting a child with additional needs. Previous research on the perspective of parents of autistic children has found that limited understanding and an absence of obvious physical features of disability are major contributing factors to their experience of stigma [23, 26, 38]. Consistent with previous autism literature, experiences of affiliate stigma appeared to be amplified in parents of children with behaviour challenges, which they reported being blamed for [25, 26, 38–40]. The perception of parents of PDA children as lenient is likely an outcome of their described adapted parenting approach. Parents reported providing their child more control than is expected by traditional perspectives of parent–child relationships, as they understood that control helped their anxious child to regulate their emotions.

Prior to learning about PDA, parents reported being advised to persist with traditional behaviour management approaches for their PDA children. The limited effectiveness of these approaches created self-doubt for parents who were parenting against their intuition. However, parents described a “light-bulb moment” in learning about PDA, which had been previously reported as a key moment in the journey of families with PDA children [27]. With more understanding about their PDA child, parents were more assured in affording their child autonomy and control to regulate their emotions. The reported effectiveness of this adapted parenting approach is consistent with Stuart et al.’s [3] explanatory model, which proposed that demand avoidant behaviours are driven by an anxious need to be in control and a strong intolerance of uncertainty. Parent’s reported experience of parenting is consistent with previous suggestions that reinforcement-based strategies such as punishment and reward are dysregulating for PDA children [7, 17]. Importantly for parents and service providers, strategies that are usually beneficial for the emotion regulation of autistic children, such as structure and routine, may be perceived by PDA children as controlling and anxiety-inducing [1]. Therefore, it is vitally important to consider the presence of PDA traits in intervention planning for autistic children.

The reported experiences of parents in this study suggest that an adapted parenting approach is needed for PDA children. Consistent with previous suggestions, a low demand, low arousal parenting approach appears to be more effective [7, 41]. An alternative model of care with a similar ethos and growing evidence base is Collaborative and Proactive Solutions (CPS; [42, 43]. The CPS model proposes that behaviour challenges occur when the demands and expectations placed on the child exceed their capacity to respond adaptively. This approach aims to collaboratively solve underlying problems with children, including accommodating skill-deficits, and decreasing demands, rather than attempting to modify their behaviour [43]. Recent evidence suggests that CPS is as efficacious as traditional parenting interventions aimed at modifying behaviour for Australian youth with behaviour challenges [44]. Additionally, CPS may hold additional benefits regarding parent–child interactions and children’s skill enhancement [43], and appears to be a preferred intervention for parents of autistic children with behaviour challenges [45]. Whilst the efficacy of CPS for PDA children would need to be explored, it appears to be a promising model of care.

As parents understood their PDA child and were able to accommodate their needs for autonomy and control, they reported an extreme reliance on them to co-regulate their child’s anxiety. One parent likened their support for their PDA child to being their child’s “wheelchair” (Lisa). This greater than expected reliance on parents for co-regulation was coupled with a reported difficulty in managing their dysregulated PDA child’s behaviour. Some parents reported experiencing violence from their child when they would become dysregulated by demands. This challenging dynamic could lead to parents avoiding potentially demanding social environments to help their child to regulate their anxiety. Parents spoke of staying home in the “home bubble” (Sharon), which is consistent with previous research that found higher levels of escape-avoidance coping in parents of autistic children [46, 47]. Parents in this study also coped through respite, gratitude, acceptance, hopefulness, and taking action.

Parenting a PDA child was reported to be a demanding and isolating experience. Isolation is a commonly reported experience for parents of autistic children [25, 48], attributed to both the rejection of others and their own avoidance [49, 50]. This dual contribution towards feelings of isolation was also apparent for parents of PDA children. However, their experience of isolation appeared to be complicated by often needing to cancel plans to attend to their anxious child’s needs for control and co-regulation. In comparison, the autistic preference for predictability may favour parents of autistic children being able to organise and seek social support that fits within the family’s routine. What appeared to buffer against feelings of isolation was the communal support from other parents of PDA children. There was an implicit understanding amongst parents with a shared experience, which helps parents to feel validated and connected [51, 52]. The value of parental support groups to parents of PDA children is an important consideration for service providers, who may facilitate families’ access and engagement.

A burden of responsibility for caregiving was also reported by parents of PDA children. This appeared to be exasperated by feelings of isolation which is consistent with existing autism research [53–55]. Family burden is a well-researched concept, defined as the personal suffering of a parent because of their family member’s illness [56]. The research literature distinguishes between objective burden, which refers to practical problems such as constraints on relationships, work, and finances, and subjective burden, which refers to parents’ emotional response [54, 57]. Parents of PDA children in this study described experiences of both objective and subjective burden. Consistent with the objective burden experienced by parents of autistic children [58], the demands of parenting a PDA child created practical problems for parents, limiting their capacity to work and maintain relationships. Parents also spoke of their difficulty attending to the competing needs of other family members, such as spending time with their non-PDA children or their partner. Another reported burden for parents was their role as advocates for their PDA children. The practical and emotional burden of advocacy had been previously described by parents of autistic children [58]. However, this burden is likely to be more pertinent for parents of PDA children, as research, understanding, and recognition of PDA is only beginning to develop (see [10–12, 21]).

Parents’ subjective burden was underlined by significant reported distress, anxiety, and exhaustion. Existing autism literature has established the contribution of caregiving burden to the elevated risk of psychopathology for parents of autistic children [54, 59, 60]. The resultant reports of stress from the demands of parenting in this study were consistent with previous research on autism and PDA [18, 61]. There is a body of research that has highlighted the importance of parents’ internal representations of their child’s diagnosis, on parental wellbeing and parent–child attachment [62–64]. Parents classified as having unresolved narratives about their child’s diagnosis often appear stuck in grief, and have difficulties with providing sensitive caregiving [62]. Unresolved parents also suffer from poorer mental and physical wellbeing [64]. High reported distress by parents in this study may be due, in part, to difficulties associated with resolving their child’s diagnosis. Resolution may be further complicated in this cohort by diagnostic uncertainty around PDA, limited knowledge, and subsequent reported experiences of feeling misunderstood.

Importantly, parents articulated their nuanced and complex emotional experience in this study, marking a key strength of qualitative research. Parents shared feelings of resentment towards PDA and their PDA child, and guilt and shame about having these unwanted and taboo feelings. Previously, parents of autistic children’s feelings of resentment appeared to be restricted to the impact of caregiving burden on parents’ careers [65]. However, in this study, parents spoke of resenting the social and emotional impacts of their challenging parenting experiences, and the thanklessness of their dedication towards their child. This dedication contributed to feelings of loss for parents who reflected on what they willingly sacrificed to care for their PDA child. Parents also grieved their expectations of a typical parent–child relationship, which is consistent with previously identified feelings of unexpected child loss [66].

The challenging and complex experiences that parents of PDA children described in this study should inform clinical practice. Parents spoke of their difficulty absorbing and implementing therapeutic interventions and recommendations, due to stress and exhaustion. Parental capacity to support their child is an important aspect of treatment effectiveness for autistic children [58, 67]. The importance of parental self-regulation is amplified by parent reports of a greater than expected need for co-regulation with their PDA children. Practitioners should consider the importance of parental emotion coaching and co-regulation for autistic children with behaviour challenges [68, 69], as autistic children often have emotion regulation difficulties [70]. Parents in this study had a unique grasp on the difficulties their PDA child faced. They demonstrated clear attempts to understand their child’s internal mental states, so that they were able to empathically support them. Psychoeducation on PDA, anxiety, and emotion regulation may facilitate parents’ mentalisation of their child to foster understanding and parental co-regulation. When professionals make attempts to understand their PDA child and adopt a non-judgemental and non-blaming approach, parents feel more positive about receiving support [27].

### Strengths and limitations

This study contributed to the emergent PDA literature by illuminating the experience of mothers of PDA children in an Australian context. The in-depth exploration of their parenting experience provided a rich, nuanced, and detailed understanding of their challenges and their relationship with their child. The current study gave voice to mothers who are advocates for their PDA children, speaking about their perspectives on what would be helpful for their families.

The interpretivist perspective adopted in this research limits its generalisability, as its aim was to explore participants’ lived experience. The study was limited in its recruitment of female-only participants. The bias towards recruiting mothers is likely to reflect their greater caregiving roles with autistic children [65]. Although there may be similarity in experience between mothers and fathers of PDA children, this study does not speak to the unique experience of fathers [71, 72]. Demographic data on participants’ cultural backgrounds was not collected and so cross-cultural differences in experiences between participants could not be explored. Information on the neurotype of the parents, and the child’s age at diagnosis, were also not collected, and could have enabled further exploration and comparison of participants’ experiences.

### Future directions

One of the key findings of this study was the need for more awareness, understanding and recognition of PDA [18], as this is beneficial for parents and their children in receiving appropriate interventions, reducing stigma, and increasing acceptance. Whilst the research literature on PDA is emerging, debate circulates about the validity of PDA diagnostic classification (see [6, 16, 73]). This debate may detract from the individual needs of families with children that identify with anxiety-related demand avoidance [18]. Despite the many challenges associated with PDA, there is extremely limited research focused on treatment [21]. The findings of this study suggest that a flexible and informed approach to service provision is required for this population. However, future research should evaluate the efficacy of collaborative and low demand interventions.

Future research may consider evaluating mental health outcomes and associated interventions for parents of PDA children. Systematic evaluation of parenting stress is warranted in this cohort, and future research may wish to explore the impact of resolution of the child’s diagnosis on parental wellbeing and attachment relationship quality in parents of children with a PDA profile (see [62–64]). There was a considerable portion of participants who did not respond to offers or attend their interview. Future research on this population should consider the impact of parenting burden on recruitment, and explore possibilities for supporting research participation. The unique experience of fathers of PDA children should also be explored, as well as cross-cultural differences in experiences of PDA. Future research may also wish to consider the impact of PDA on siblings, as research shows adapted relationships and impacts on emotions and behaviours for siblings of people with disability [74].

Future research may wish to directly compare the experiences of parents of autistic children with a PDA profile, with the experiences of parents of autistic children without PDA and typically developing children. This could help to distinguish aspects of parenting that are unique to parenting a PDA child. The importance of lived experience in research, policy, and intervention development for autistic people cannot be overlooked [33, 34]. Thus, the PDA community should continue to be consulted as key stakeholders in future research directions. Exposure of lived experience to mainstream academic and social platforms should be facilitated, to de-stigmatise PDA through influencing understanding, attitudes, and perceptions.

## Conclusion

This study illuminated the challenging experiences of parents of autistic children with a PDA profile and their complex and special relationships with their children. There is a need for further recognition, understanding, and acceptance of PDA, and for flexible, informed, and appropriate support for PDA children and their families.

## Supplementary Information

Below is the link to the electronic supplementary material.

**Supplementary file 1**.

## Data Availability

To protect participant privacy, research data is not publicly available, although anonymised data can be made accessible upon request.

## References

[1] Newson EL, Le Marechal K, David C. Pathological demand avoidance syndrome: a necessary distinction within the pervasive developmental disorders. Arch Dis Child. 2003;88(7):595–600. 10.1136/adc.88.7.595. 12818906 10.1136/adc.88.7.595 PMC1763174

[2] Woods R. Rational (Pathological) demand avoidance: as a mental disorder and an evolving social construct. Routledge Int Handbook Crit Autism Stud. 2022. 10.4324/9781003056577-7.

[3] Stuart L, Grahame V, Honey E, Freeston M. Intolerance of uncertainty and anxiety as explanatory frameworks for extreme demand avoidance in children and adolescents. Child Adolesc Mental Health. 2020;25(2):59–67. 10.1111/camh.12336. 10.1111/camh.12336 32307839

[4] O’Nions E, Viding E, Greven CU, Ronald A, Happé F. Pathological demand avoidance: exploring the behavioural profile. Autism. 2014;18(5):538–44. 10.1177/1362361313481861. 24104509 10.1177/1362361313481861

[5] O’Nions E, Viding E, Floyd C, Quinlan E, Pidgeon C, Gould J, Happé F. Dimensions of difficulty in children reported to have an autism spectrum diagnosis and features of extreme/‘pathological’ demand avoidance. Child Adolesc Mental Health. 2018;23(3):220–7. 10.1111/camh.12242. 10.1111/camh.12242 32677301

[6] Green J, Absoud M, Grahame V, Malik O, Simonoff E, Le Couteur A, Baird G. Pathological demand avoidance: symptoms but not a syndrome. Lancet Child Adolesc Health. 2018;2(6):455–64. 10.1016/S2352-4642(18)30044-0. 30169286 10.1016/S2352-4642(18)30044-0

[7] O’Nions E, Eaton J. Extreme/‘pathological’demand avoidance: an overview. Paediatr Child Health. 2020;30(12):411–5. 10.1016/j.paed.2020.09.002.

[8] American Psychiatric Association. Diagnostic and statistical manual of mental disorders. 5th ed. Text revision. 2022. 10.1176/appi.books.9780890425787

[9] World Health Organization. International statistical classification of diseases and related health problems. 11th ed. 2019. https://icd.who.int/

[10] Guldberg K, Bradley R, Wittemeyer J, Briscombe C, Phillips C, Jones G. Good autism practice: Practitioner guide. Autism Education Trust. 2019. https://www.egfl.org.uk/sites/default/files/Services_for_children/SEND/Practitioner-Guidelines_nov_19.pdf

[11] National Autistic Society. Demand Avoidance. https://www.autism.org.uk/advice-and-guidance/topics/behaviour/demand-avoidance. Accessed 15 Jul 2023.

[12] Whitehouse AJ, Evans K, Eapen V, Wray J. A national guideline for the assessment and diagnosis of autism spectrum disorders in Australia. Brisbane, Australia: Autism Cooperative Research Centre (CRC). 2018. https://www.autismcrc.com.au/access/sites/default/files/resources/National_Guidline_Response_to_Consultation_Public_Submissions.pdf

[13] Gillberg C, Gillberg IC, Thompson L, Biskupsto R, Billstedt E. Extreme (“pathological”) demand avoidance in autism: a general population study in the Faroe Islands. Eur Child Adolesc Psychiatry. 2015;24:979–84. 10.1007/s00787-014-0647-3. 25398390 10.1007/s00787-014-0647-3

[14] PDA Society. What is demand avoidance?. https://www.pdasociety.org.uk/what-is-pda-menu/what-is-demand-avoidance/ Accessed 11 Jul 2023.

[15] PDA Society. Diagnosing PDA in children https://www.pdasociety.org.uk/i-am-a-parent-carer/resources/diagnosing-pda/ Accessed 11 Jul 2023.

[16] Woods R. Demand avoidance phenomena: circularity, integrity and validity–A commentary on the 2018 National Autistic Society PDA Conference. Good Autism Practice. 2019;20(2):28–40. https://openresearch.lsbu.ac.uk/item/8qx90

[17] Christie P, Duncan M, Healy Z, Fidler R. Understanding pathological demand avoidance syndrome in children: a guide for parents, teachers and other professionals. Jessica Kingsley Publishers; 2012.

[18] Doyle A, Kenny N. Mapping experiences of pathological demand avoidance in Ireland. J Res Spec Educ Needs. 2023;23(1):52–61. 10.1111/1471-3802.12579.

[19] Running A, Jata-Hall D. Parental blame and the PDA profile of Autism*.* PDA society. 2023. https://www.pdasociety.org.uk/wp

[20] Truman C, Crane L, Howlin P, Pellicano E. The educational experiences of autistic children with and without extreme demand avoidance behaviours. Int J Incl Educ. 2024;28(1):57–77. 10.1080/13603116.2021.1916108.

[21] Kildahl AN, Helverschou SB, Rysstad AL, Wigaard E, Hellerud JM, Ludvigsen LB, Howlin P. Pathological demand avoidance in children and adolescents: a systematic review. Autism. 2021;25(8):2162–76. 10.1177/13623613211034382. 34320869 10.1177/13623613211034382

[22] Makino A, Hartman L, King G, Wong PY, Penner M. Parent experiences of autism spectrum disorder diagnosis: a scoping review. Rev J Autism Develop Disorders. 2021. 10.1007/s40489-021-00237-y.

[23] Ooi KL, Ong YS, Jacob SA, Khan TM. A meta-synthesis on parenting a child with autism. Neuropsychiatric Disease Treat. 2016. 10.2147/NDT.S100634. 10.2147/NDT.S100634 PMC4827600 27103804

[24] Corcoran J, Berry A, Hill S. The lived experience of US parents of children with autism spectrum disorders: a systematic review and meta-synthesis. J Intellect Disabil. 2015;19(4):356–66. 10.1177/1744629515577876. 25819433 10.1177/1744629515577876

[25] Gray DE. Perceptions of stigma: The parents of autistic children. Sociol Health Illn. 1993;15(1):102–20. 10.1111/1467-9566.ep11343802.

[26] Gray DE. ‘Everybody just freezes. Everybody is just embarrassed’: felt and enacted stigma among parents of children with high functioning autism. Soc Health Illness. 2002;24(6):734–49. 10.1111/1467-9566.00316.

[27] Gore Langton E, Frederickson N. Parents’ experiences of professionals’ involvement for children with extreme demand avoidance. Int J Develop Disabilit. 2018;64(1):16–24. 10.1080/20473869.2016.1204743. 10.1080/20473869.2016.1204743 PMC8115513 34141287

[28] Smith JA, Larkin M, Flowers P. Interpretative phenomenological analysis: theory, method and research. 2nd ed. Sage. 2022.

[29] Smith JA, Osborn M. Interpretative phenomenological analysis. In Smith JA, editors. Qualitative psychology: a practical guide to methods. Sage; 2003. pp. 25–53.

[30] O’Nions E, Christie P, Gould J, Viding E, Happé F. Development of the ‘Extreme demand avoidance questionnaire’(EDA-Q): preliminary observations on a trait measure for pathological demand avoidance. J Child Psychol Psychiatry. 2014;55(7):758–68. 10.1111/jcpp.12149. 24117718 10.1111/jcpp.12149

[31] Tufford L, Newman P. Bracketing in qualitative research. Qual Soc Work. 2012;11(1):80–96. 10.1177/1473325010368316.

[32] Dodgson JE. Reflexivity in qualitative research. J Hum Lact. 2019;35(2):220–2. 10.1177/0890334419830990. 30849272 10.1177/0890334419830990

[33] Benevides TW, Shore SM, Palmer K, Duncan P, Plank A, Andresen ML, Caplan R, Cook B, Gassner D, Hector BL, Morgan L. Listening to the autistic voice: mental health priorities to guide research and practice in autism from a stakeholder-driven project. Autism. 2020;24(4):822–33. 10.1177/1362361320908410. 32429818 10.1177/1362361320908410 PMC7787673

[34] Milton D, Bracher M. Autistics speak but are they heard. Med Soc Online. 2013;7(2):61–9.

[35] Wing L, Leekam SR, Libby SJ, Gould J, Larcombe M. The diagnostic interview for social and communication disorders: Background, inter-rater reliability and clinical use. J Child Psychol Psychiatry. 2002;43(3):307–25. 10.1111/1469-7610.00023. 11944874 10.1111/1469-7610.00023

[36] Goffman E. Stigma: Notes on the management of spoiled identity. Simon and Schuster; 2009.

[37] Larson JE, Corrigan P. The stigma of families with mental illness. Acad Psychiatry. 2008;32:87–91. 10.1176/appi.ap.32.2.87. 18349326 10.1176/appi.ap.32.2.87

[38] Salleh NS, Abdullah KL, Yoong TL, Jayanath S, Husain M. Parents’ experiences of affiliate stigma when caring for a child with autism spectrum disorder (ASD): a meta-synthesis of qualitative studies. J Pediatr Nurs. 2020;55:174–83. 10.1016/j.pedn.2020.09.002. 32957021 10.1016/j.pedn.2020.09.002

[39] Chan KK, Lam CB. Trait mindfulness attenuates the adverse psychological impact of stigma on parents of children with autism spectrum disorder. Mindfulness. 2017;8:984–94. 10.1007/s12671-016-0675-9.

[40] Werner S, Shulman C. Does type of disability make a difference in affiliate stigma among family caregivers of individuals with autism, intellectual disability or physical disability? J Intellect Disabil Res. 2015;59(3):272–83. 10.1111/jir.12136. 24761747 10.1111/jir.12136

[41] Diekman A. Low-demand Parenting: dropping demands, restoring calm, and finding connection with your uniquely wired child. Jessica Kingsley Publishers; 2023.

[42] Greene R. The explosive child: A new approach for understanding and parenting easily frustrated, “chronically inflexible” children. Harper Collins Publishers. 1998.

[43] Greene R, Winkler J. Collaborative & proactive solutions (CPS): a review of research findings in families, schools, and treatment facilities. Clin Child Fam Psychol Rev. 2019;22(4):549–61. 10.1007/s10567-019-00295-z. 31240487 10.1007/s10567-019-00295-z

[44] Murrihy RC, Drysdale SA, Dedousis-Wallace A, Rémond L, McAloon J, Ellis DM, Halldorsdottir T, Greene RW, Ollendick TH. Community-delivered collaborative and proactive solutions and parent management training for oppositional youth: a randomized trial. Behav Ther. 2023;54(2):400–17. 10.1016/j.beth.2022.10.005. 36858768 10.1016/j.beth.2022.10.005

[45] Tschida JE, Maddox BB, Bertollo JR, Kuschner ES, Miller JS, Ollendick TH, Greene RW, Yerys BE. Caregiver perspectives on interventions for behavior challenges in autistic children. Res Autism Spect Disord. 2021;81: 101714. 10.1016/j.rasd.2020.101714.

[46] Dunn ME, Burbine T, Bowers CA, Tantleff-Dunn S. Moderators of stress in parents of children with autism. Community Ment Health J. 2001;37:39–52. 10.1023/A:1026592305436. 11300666 10.1023/a:1026592305436

[47] Pisula E, Kossakowska Z. Sense of coherence and coping with stress among mothers and fathers of children with autism. J Autism Dev Disord. 2010;40:1485–94. 10.1007/s10803-010-1001-3. 20361246 10.1007/s10803-010-1001-3

[48] Kocabiyik OO, Fazlioglu Y. Life stories of parents with Autistic children. J Educat Train Stud. 2018;6(3):26–37. 10.11114/jets.v6i3.2920.

[49] Kinnear SH, Link BG, Ballan MS, Fischbach RL. Understanding the experience of stigma for parents of children with autism spectrum disorder and the role stigma plays in families’ lives. J Autism Dev Disord. 2016;46:942–53. 10.1007/s10803-015-2637-9. 26659549 10.1007/s10803-015-2637-9

[50] Mazumder R, Thompson-Hodgetts S. Stigmatization of children and adolescents with autism spectrum disorders and their families: a scoping study. Rev J Autism Develop Disord. 2019;6:96–107. 10.1007/s40489-018-00156-5.

[51] Batchelor M, Maguire S, Shearn J. “They just get it” an exploration of father’s experiences and perceptions of a support group for men caring for children with disabilities and/or developmental delay. J Appl Res Intellect Disabil. 2021;34(1):263–73. 10.1111/jar.12804. 33047415 10.1111/jar.12804

[52] Lee JD, Terol AK, Yoon CD, Meadan H. Parent-to-parent support among parents of children with autism: a review of the literature. Autism. 2024;28(2):263–75. 10.1177/13623613221146444. 36588317 10.1177/13623613221146444

[53] Ekas NV, Lickenbrock DM, Whitman TL. Optimism, social support, and well-being in mothers of children with autism spectrum disorder. J Autism Dev Disord. 2010;40:1274–84. 10.1007/s10803-010-0986-y. 20195734 10.1007/s10803-010-0986-y

[54] Picardi A, Gigantesco A, Tarolla E, Stoppioni V, Cerbo R, Cremonte M, Alessandri G, Lega I, Nardocci F. Parental burden and its correlates in families of children with autism spectrum disorder: a multicentre study with two comparison groups. Clin Pract Epidemiol Mental Health CP & EMH. 2018;14:143–76. 10.2174/1745017901814010143. 30158998 10.2174/1745017901814010143 PMC6080067

[55] Zaidman-Zait A, Mirenda P, Duku E, Vaillancourt T, Smith IM, Szatmari P, Bryson S, Fombonne E, Volden J, Waddell C, Zwaigenbaum L. Impact of personal and social resources on parenting stress in mothers of children with autism spectrum disorder. Autism. 2017;21(2):155–66. 10.1177/1362361316633033. 27091948 10.1177/1362361316633033

[56] Schene AH. Objective and subjective dimensions of family burden: towards an integrative framework for research. Soc Psychiatry Psychiatr Epidemiol. 1990;25:289–97. 10.1007/BF00782883. 2291131 10.1007/BF00782883

[57] Magliano L, Fiorillo A, DeRosa C, Malangone C, Maj M. National Mental Health Project Working Group Family burden in long-term diseases: a comparative study in schizophrenia vs. physical disorders. Soc Sci Med. 2005;61(2):313–22. 10.1016/j.socscimed.2004.11.064. 15893048 10.1016/j.socscimed.2004.11.064

[58] Karst JS, Van Hecke AV. Parent and family impact of autism spectrum disorders: a review and proposed model for intervention evaluation. Clin Child Fam Psychol Rev. 2012;15:247–77. 10.1007/s10567-012-0119-6. 22869324 10.1007/s10567-012-0119-6

[59] Hodge D, Hoffman CD, Sweeney DP. Increased psychopathology in parents of children with autism: genetic liability or burden of caregiving? J Dev Phys Disabil. 2011;23:227–39. 10.1007/s10882-010-9218-9.

[60] Magallon-Neri E, Vila D, Santiago K, Garcia P, Canino G. The prevalence of psychiatric disorders and mental health services utilization by parents and relatives living with individuals with autism spectrum disorders in Puerto Rico. J Nerv Ment Dis. 2018;206(4):226–30. 10.1097/NMD.0000000000000760. 29112530 10.1097/NMD.0000000000000760

[61] Hayes SA, Watson SL. The impact of parenting stress: a meta-analysis of studies comparing the experience of parenting stress in parents of children with and without autism spectrum disorder. J Autism Dev Disord. 2013;43:629–42. 10.1007/s10803-012-1604-y. 22790429 10.1007/s10803-012-1604-y

[62] Marvin RS, Pianta RC. Mothers’ reactions to their child’s diagnosis: relations with security of attachment. J Clin Child Psychol. 1996;25(4):436–45. 10.1207/s15374424jccp2504_8.

[63] Pianta RC, Marvin RS, Britner PA, Borowitz KC. Mothers’ resolution of their children’s diagnosis: Organized patterns of caregiving representations. Infant Mental Health J. 1996;17(3):239–56. 10.1002/(SICI)1097-0355(199623)17:3%3c239::AID-IMHJ4%3e3.0.CO;2-J.

[64] Sher-Censor E, Shahar-Lahav R. Parents’ resolution of their child’s diagnosis: a scoping review. Attach Hum Dev. 2022;24(5):580–604. 10.1080/14616734.2022.2034899. 35156548 10.1080/14616734.2022.2034899

[65] Gray DE. Gender and coping: the parents of children with high functioning autism. Soc Sci Med. 2003;56(3):631–42. 10.1016/S0277-9536(02)00059-X. 12570979 10.1016/s0277-9536(02)00059-x

[66] Fernańdez-Alcántara M, García-Caro MP, Pérez-Marfil MN, Hueso-Montoro C, Laynez-Rubio C, Cruz-Quintana F. Feelings of loss and grief in parents of children diagnosed with autism spectrum disorder (ASD). Res Dev Disabil. 2016;55:312–21. 10.1016/j.ridd.2016.05.007. 27235768 10.1016/j.ridd.2016.05.007

[67] Burrell TL, Borrego J Jr. Parents’ involvement in ASD treatment: what is their role? Cogn Behav Pract. 2012;19(3):423–32. 10.1016/j.cbpra.2011.04.003.

[68] Ting V, Weiss JA. Emotion regulation and parent co-regulation in children with autism spectrum disorder. J Autism Dev Disord. 2017;47:680–9. 10.1007/s10803-016-3009-9. 28070784 10.1007/s10803-016-3009-9 PMC5352765

[69] Wilson BJ, Berg JL, Zurawski ME, King KA. Autism and externalizing behaviors: buffering effects of parental emotion coaching. Res Autism Spect Disord. 2013;7(6):767–76. 10.1016/j.rasd.2013.02.005.

[70] Mazefsky CA, Herrington J, Siegel M, Scarpa A, Maddox BB, Scahill L, White SW. The role of emotion regulation in autism spectrum disorder. J Am Acad Child Adolesc Psychiatry. 2013;52(7):679–88. 10.1016/j.jaac.2013.05.006. 23800481 10.1016/j.jaac.2013.05.006 PMC3719386

[71] Burrell A, Ives J, Unwin G. The experiences of fathers who have offspring with autism spectrum disorder. J Autism Dev Disord. 2017;47:1135–47. 10.1007/s10803-017-3035-2. 28132126 10.1007/s10803-017-3035-2 PMC5357286

[72] Lashewicz BM, Shipton L, Lien K. Meta-synthesis of fathers’ experiences raising children on the autism spectrum. J Intellect Disabil. 2019;23(1):117–31. 10.1177/1744629517719347. 28705095 10.1177/1744629517719347

[73] Moore A. Pathological demand avoidance: What and who are being pathologised and in whose interests? Global Stud Childhood. 2020;10(1):39–52. 10.1177/2043610619890070.

[74] Levante A, Martis C, Del Prete CM, Martino P, Primiceri P, Lecciso F. Siblings of persons with disabilities: a systematic integrative review of the empirical literature. Clin Child Fam Psychol Rev. 2024. 10.1007/s10567-024-00502-6. 39414751 10.1007/s10567-024-00502-6 PMC11885339

