# Supplementary material for: The experience of mothers of autistic children with a pathological demand avoidance profile: an interpretative phenomenological analysis
Source: Discov Ment Health. 2025 Jan 20;5(1):5. doi: 10.1007/s44192-025-00127-3 (PMC11747059; doi:10.1007/s44192-025-00127-3)
Supplement: Supplementary file 1 [file 44192_2025_127_MOESM1_ESM.docx]

Supplementary Material

"The Experience of Mothers of Autistic Children with Pathological Demand Avoidance: An Interpretative Phenomenological Analysis "

Semi-Structured Interview Guide

**Background Information and Rapport Building:**

Can you tell me about your family?

Can you tell me about your child?

How would you describe them?

What was the impact of their autism diagnosis?

What made you think that your autistic child had pathological demand avoidance?

What is your understanding of pathological demand avoidance?

**The Caregiver Experience:**

What is it like to care for a child with pathological demand avoidance?

What effect has your child’s pathological demand avoidance had on you?

What effect do you think it has had on your wellbeing?

How has your child’s pathological demand avoidance affected your relationship with them?

What is it like for you when your child engages in demand avoidant behaviours?

What does it feel like?

How has pathological demand avoidance affected your family?

What impact has it had on the other children?

How has it affected the relationship between you and your partner?

When you worry about your child, what do you worry about the most?

What kind of situations make you feel this way?

What do you think you need as the parent/caregiver of a pathological demand avoidant child?

What would you like other people to know about pathological demand avoidance?

**Question Prompts:**

Can you tell me more about that?

What was that like for you?
